# Effects of An Acute Physical Activity Break on Test Anxiety and Math Test Performance

**DOI:** 10.3390/ijerph17051523

**Published:** 2020-02-27

**Authors:** Myrto F. Mavilidi, Kim Ouwehand, Nicholas Riley, Paul Chandler, Fred Paas

**Affiliations:** 1 Priority Research Centre for Physical Activity and Nutrition, School of Education, University of Newcastle, Callaghan, NSW 2308, Australia; Nicholas.Riley@newcastle.edu.au; 2 Early Start, Faculty of Social Sciences, University of Wollongong, Keiraville, NSW 2522, Australia; chandler@uow.edu.au (P.C.); paas@essb.eur.nl (F.P.); 3 Department of Psychology, Education, and Child Studies, Erasmus University Rotterdam, 3062 PA Rotterdam, The Netherlands; ouwehand@essb.eur.nl

**Keywords:** physical activity break, test anxiety, academic performance, primary school children

## Abstract

(1) Background: Test anxiety has been found to negatively affect students’ mental health and academic performance. A primary explanation for this is that anxiety-related thoughts occupy working memory resources during testing that cannot be used for test-related processes (such as information retrieval and problem-solving). The present intervention study investigated whether physical activity could decrease anxiety levels and improve math test performance in sixth-grade children. (2) Methods: Sixty-eight children of 11–12 years from two primary schools in New South Wales, Australia were categorised as low or high anxious from their scores on a trait-anxiety questionnaire. After this assessment, they were randomly assigned to the activity break condition, in which they had to do several physical activities of moderate intensity (e.g., star jumps) for 10 min, or the control condition, in which they played a vocabulary game for 10 min. The outcome measures were children’s anxiety levels at the beginning, during, and at the end of the test, invested mental effort, perceived task difficulty and math test performance. (3) Results: Results showed that regardless of the condition, low anxious students performed better on the math test than high anxious children. No differences were found for any of the variables between the activity break condition and the control condition. (4) Conclusions: Although test anxiety was not reduced as expected, this study showed that short physical activity breaks can be used before examinations without impeding academic performance.

## 1. Introduction

During their school years, roughly one third of elementary and secondary school children suffer from test anxiety [1,2] which impairs optimal performance, especially during high-stakes tests (e.g., [3,4,5]). Anxiety is an aversive motivational state occurring when the level of perceived threat is considered high [6]. Within the concept of anxiety, an often-made distinction is made between trait anxiety when anxiety refers to differences in personality dimensions and state anxiety when referring to anxiety as a transient mood state. Both types of anxiety hamper performance, especially during complex and attentionally demanding tasks [6,7]. Test anxiety has physiological (e.g., increased heart rate [8]), cognitive (e.g., worries [9]), behavioural and emotional effects (e.g., wish to elude from the test situations, fear of social comparison, sense of inefficacy and of being unprepared [10,11]). The worries and anxiety-related intrusive thoughts interfere with working memory functioning, impairing test performance [12]. The present study explored whether a short bout of physical activity can alleviate test anxiety and enhance test performance in sixth-grade children. The rationale behind this research question was based on a biopsychosocial and a cognitive psychology perspective. The biopsychosocial model of challenge and threat (BPS [13]) provides a physiological explanation for the positive effects of physical exercise on anxiety relief depending on the appraisal of challenge and threat. When performing a task that is self-relevant and/or of personal importance, heart rate increases. When challenged, the blood vessels respond in an adaptive manner and dilate to allow more blood flow through the body (a similar process that occurs during aerobic exercise). However, the appraisal of threat activates the body’s stress system (HPA axis: for a more detailed description of this mechanism [14]), causing arteries constrict rather than dilate, a response counteractive to the increased heart rate. We suggest that, in the situation of test anxiety, inducing vessel dilation by physical exercise (instead of cognitive appraisal), can also change the physiological threat response in a more adaptive challenge response, which can lead to stress relief and improved test performance.

An explanation from a cognitive perspective on how exercise might improve children’s cognitive performance can be found in the cognitive load theory (CLT [15,16]). This theory states that for optimal learning and performance, instructions and training should take the limitations of working memory into account. In explaining working memory, CLT uses the definition of Baddeley (2000) [17], in which working memory is a “limited capacity system allowing the temporary storage and manipulation of information necessary for such complex cognitive tasks as language comprehension, learning and reasoning” (p. 418). CLT distinguishes between three types of load: (1) intrinsic load, which is imposed by the complexity of the study material and consequently depends on the learner’s prior knowledge, (2) extraneous load, which is imposed by the way the information is presented (i.e., instructional design) and has a negative effect on learning, and (3) germane cognitive load, which is related to the cognitive resources allocated to deal with intrinsic load [18].

It can be concluded that learning will be impeded when the limited working memory capacity is overloaded, for example, when there is too much extraneous load, which can happen under conditions of high task difficulty (intrinsic load is high), badly designed instructions (extraneous load is high), and anxiety-related ruminations (germane load is low). In contrast, learning will be enhanced with instructional strategies that decrease extraneous load, thereby freeing working memory resources that can be allocated to intrinsic aspects of the learning task. For example, a conventional problem-solving strategy in which only the problem statement is presented imposes high extraneous load because all steps and outcomes need to be kept active in working memory until they lead to the solution. This process leaves little (if any) resources for learning. An effective strategy to deal with this high extraneous load is the use of worked-out examples in which all steps to the solution are given and can be studied by learners [19,20]). We suggest that physical activity might reduce the extraneous load caused by anxiety. By reducing this load, more working memory resources become available for the task. In contrast, incontrollable worries and intrusive thoughts stemming from test anxiety are believed to occupy working memory capacity, thereby reducing the learners’ resources available for learning (germane load).

Following this line of reasoning, CLT would predict that reducing the intrusive thoughts occupying working memory capacity should free capacity that can be used to improve learning. Based on a similar theoretical argument, Ramirez and Beilock (2011) [21] found that a cognitive-behavioural intervention, in which participants had to write down anxiety-related thoughts 10 min before a math test, reduced working memory load and improved test performance. In another study conducted within this theoretical framework by Mavilidi, Hoogerheide and Paas (2014) [22], it was found that when students were allowed to have a short glance (1 min) at the questions of a math test before starting to solve the problems, they performed better in a math test compared to the conventional control group.

As mentioned earlier, in the present study, we investigated the possible effect of short bouts of physical activity on test anxiety. To the best of our knowledge, research on the effects of acute physical activity on test anxiety in children is very limited. Most studies examined the long-term effects of physical activity and regular exercise on anxiety involving multiple bouts in adults.

Aerobic exercise has been shown to reduce symptoms of anxiety and depression and stress-related complaints in subclinical population [23]. A cross-sectional survey reported that only 40% of the Canadian population from 15 years and older uses exercise to cope with stress [24], even though exercise training as a stress-management treatment had been shown to reduce anxiety levels and improve lifestyle in adults [25]. Other empirical evidence attests that anxiety and depressive symptoms, prevalent in less active people, can be reduced through regular physical activity [26,27,28]. In addition, a study with female participants showed that even trait anxiety can be reduced by a physical fitness program [29]. In a 10-week exercise program trait anxiety of an experimental group, which included one aerobic and two step dance sessions per week, was compared with a control group, which did not participate in any regular physical activity program. While starting at similar levels of trait anxiety, after ten weeks, the experimental group showed lower levels of trait anxiety than the control group.

The majority of research has utilised physical exercise in clinical populations diagnosed with mental health disorders to reduce anxiety, stress and depressive symptoms, targeting mainly adults [23,27,30], but not children [31,32]. However, it cannot be ruled out that the physical activity interventions might have positive effects also as preventive strategies in the emergence and prevalence of anxiety. Importantly, if exercise can improve cognitive performance, this can have important implications for educational outcomes (i.e., cognitive and academic performance).

Nonetheless, there is strong evidence supporting the positive effects of acute exercise on children’s and youth’s cognitive performance, in particular on executive functions [33,34]. For instance, research utilising acute (i.e., single sessions) bouts of cognitive demanding physical activity has found mixed results, with some studies depicting improvements in children’s executive functions [35,36], whereas other studies reported worsening effects in children’s attention scores in the short-term [37,38]. In addition, research on active breaks mainly focusing on chronic school-based interventions found improvements on children’s on-task behaviour but conflicting results on students’ academic achievement and cognitive function [39,40]. In particular, some studies found positive [36,41,42,43], as well as adverse or negative effects [35,44].

An abundance of research suggests that repetitive exercise can alleviate stress, increase enjoyment, and foster academic achievement [45]. A common methodology of these studies was the use of multiple classroom breaks. Physical activity breaks lasting from 5 to 20 min (typically 5–10 min in duration) have been found to have positive selective effects on children’s cognitive functions (e.g., inhibition [35]; attention [44]; shifting [46]), on-task behaviour and academic achievement [41,42,47,48]. For example, a recent study integrating physical activity with language learning in primary school children for 10 min per day, twice per week for two weeks found deteriorated effects on attentional performance while children’s learning was improved after the end of the intervention [49]. The reported level of cognitive exertion did not differ between the condition that integrated physical activity with learning and the control condition.

Interestingly, recent systematic reviews showed that physically active lessons can also improve children’s academic outcomes (e.g., on-task behaviour, executive function skills, and academic achievement [40,50]). For instance, children had the highest learning outcomes and reported it as the most enjoyable way of learning compared to the usual sedentary curriculum when academic lessons were integrated with physical activity in several academic areas such as language, maths, science, or geography [49,51,52,53,54,55].

To conclude, even if there is growing evidence on the effects of regular physical activity on cognitive performance, there is still a lack of research looking into short single bouts of physical activity on test anxiety and math performance in children. The present study explored whether a short bout of physical activity can alleviate test anxiety and enhance test performance in sixth-grade children. A randomized-controlled trial was conducted to investigate the immediate effects of an acute classroom physical activity break on test anxiety and math test performance in primary school children. In the physical activity break group (experimental group), the participants were engaged in 10 min of physical activity prior to the test, whereas in the control group, the participants played a vocabulary game on the traditional blackboard for 10 min. First, it was hypothesised that the high-anxious student would perform worse than low-anxious students. Second, it was hypothesised that the activity break group would show lower anxiety levels in general and would outperform the control group in their academic performance because children would release their anxiety thoughts through physical activity.

## 2. Materials and Methods

### 2.1. Participants

The study was carried out in two primary schools in New South Wales, Australia. Participants were 68 sixth-grade children 11–12 years. The study received approval from the Human Research Ethics Committee of the University of Wollongong (No: HE15/195). Principals of independent schools were informed about the study. Upon principals’ approval, parents gave their written consent and children their oral assent. Students in each class were divided into two anxiety groups, low and high, based on their scores on the cognitive anxiety test questionnaire [56]. Within each anxiety group participants were randomly assigned to the control (*n =* 35; 21 anxious and 14 low anxious students) or the activity break condition (*n =* 33; 20 anxious and 13 low anxious students).

### 2.2. Experimental Design and Statistical Analysis

A between-subjects 2 × 2 design was used with trait anxiety (low vs. high) and condition (activity break vs. control) as between-subjects factors. The dependent variables were math test performance, invested mental effort, perceived task difficulty, and a repeated measure for state anxiety measured at three points in time (i.e., at the beginning, during, and at the end of the lesson).

The IBM SPSS Statistics 25 software was used for the analyses. To examine the role of condition on anxiety, we conducted a mixed ANOVA with time of measuring test anxiety (at the start, during, and at the end) as within-subjects factor, and condition and anxiety level as between-subjects factors. To inspect the effect of condition and anxiety on math performance, a two-way analysis of covariance (ANCOVA) was conducted, with test strategy (activity break group vs. vocabulary game) and anxiety level (low vs high) as between-subjects factors, and previous teachers’ grade as the covariate. Regarding perceived task difficulty, a two-way ANOVA was conducted with condition and anxiety level as between-subjects factors.

### 2.3. Materials

The materials used were adopted from the study of Mavilidi et al. (2014) [22]. One booklet was prepared for each student containing the cognitive anxiety test questionnaire, the math test, questions about test anxiety, children’s mental effort, and perceived task difficulty.

#### 2.3.1. Test Anxiety

Test anxiety was measured using the Cognitive Anxiety test Questionnaire [57] one week before the beginning of the intervention, and using self-reported responses. The questionnaire included 27 items measuring trait anxiety on a 4-point item scale ranging from 1, not typical for me, to 4, very typical of me. The minimum score that a child could receive was 27, while the maximum was 108. This questionnaire has been shown to have high internal consistency (α = 0.86) and was used to categorise students to low and high anxious.

At the end of the math test questions, students rated their anxiety levels on a 1 to 9-point Likert scale ranging from 1, very low anxious, to 9, very high anxious regarding how they felt at the beginning, during, and at the end of the test.

#### 2.3.2. Math Test

The test consisted of 7 open-ended test problems selected from a sample of the California standard tests (STAR, nd: http://www.cde.ca.gov/ta/tg/sr/documents/cstrtqmath6.pdf) and test problems created by the Dutch National Institute for Educational Measurement (CITO, nd) for the end of primary school test (www.eindtoets.nl). The mathematical content included appropriate topics from the Australian syllabus that students had been studying throughout the year such as combined problems, and/or simple algebraic equations. Students earned 1 point for each correct answer (minimum = 0, maximum = 7). The level of complexity for the test was considered high, due to the number of different interacting information elements co-occurring in the problems [57].

#### 2.3.3. Perceived Task Difficulty

Children rated the level of perceived task difficulty at the end of each test problem on a 9-point item scale ranging from 1, very very low task difficulty, to 9, very very high task difficulty, adjusted from Sweller et al. (2011) [18].

#### 2.3.4. Perceived Mental Effort

Children rated the perceived amount of invested mental effort at the end of each test problem on a 9-point symmetrical category scale ranging from 1, very very low mental effort, to 9, very very high mental effort [58,59].

### 2.4. Procedure

The study was conducted in classrooms assigned to the different conditions by the lead author, with the role of teachers being merely supportive. One week before the math test, children were administered the test anxiety questionnaire. Based on their scores on the test anxiety questionnaire, students were categorised into low and high-test anxious. Within the low and high anxious groups, students were randomly assigned to either the physical activity condition, or the control condition.

At the day of the test, classrooms were re-arranged, where necessary, so that children were keeping a safe distance from each other while performing the physical activities. The instructor gave directions for the math test and the mental effort, task difficulty and anxiety scales, lasting for 5 min, at the beginning of the test. Students were not allowed to ask further questions, clarifications, or explanations during the test. In the activity break condition, children performed a 10-minute bout of physical activity (e.g., push-ups, star jumps, penguin movements, burpees, and running on the spot). In the control condition, children played the hangman game the on the school’s whiteboard for 10 minutes. Activities in both conditions were performed as a group.

**Figure 1:**
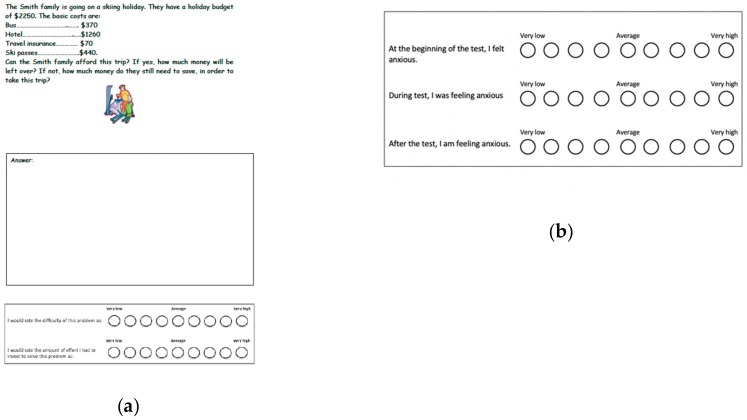
(**a**) Example of math test question and ratings for task difficulty and mental effort. (**b**) Ratings for anxiety at the beginning, during, and end of math test.

Afterwards, children completed a math test within 20 minutes. At the end of each test problem, children indicated on the rating scales how complex they perceived the problem and how much mental effort they had invested in it (see Figure 1). Children indicated their anxiety level at the beginning, during, and at the end of the test on the rating scale provided in the test booklet. In order to induce anxiety, children were told that they were matched with a fellow student from the other class who had already sat the test, creating a team consisting of two students. This strategy was similar to previous studies [21,22]. Ecological validity was ensured by assessing students during normal classroom conditions. Children were awarded with a gift voucher of $7 for their participation, without being aware of the monetary reward before the end of the assessment.

## 3. Results

### 3.1. Anxiety at the Beginning, During, and at the End of the Test

Mauchly’s test of sphericity indicated that the assumption of sphericity was violated, χ^2^(2) = 7.78, *p* = 0.020. The Greenhouse–Geisser was *ε >* 0.75, therefore we used the Huynh–Feldt correction [60]. Results revealed a significant main effect of anxiety level on the time of measuring test anxiety, *F*(1, 70) = 7.71, *p* = 0.007, η_p_^2^ = 0.12, and a significant interaction between anxiety level and time, *F*(1.93, 135.25) = 3.72, *p* = 0.028, η_p_^2^ = 0.05. However, the main effects of condition, *F*(1, 70) = 0.46, *p* = 0.498, η_p_^2^ < 0.01, and time, *F*(1.93, 135.25) = 1.47, *p* = 0.233, η_p_^2^ = 0.02, were not significant. Also, the interactions between condition and anxiety level, *F*(1, 70) = 0.13, *p* = 0.498, η_p_^2^ < 0.01, time and condition, *F*(1.93, 135.25) = 0.16, *p* = 0.842, η_p_^2^ < 0.01, and time, condition and anxiety level, *F*(1.93, 135.25) = 0.19, *p* = 0.818, η_p_^2^ < 0.01, were not significant. Polynomial contrasts tests revealed that there was a quadratic relationship for the interaction between children’s anxiety level and time, *F*(1, 70) = 7.62, *p* = 0.007, η_p_^2^ = 0.10. High anxious children were more anxious at the beginning and during the test, but their anxiety dropped at the end of the test. Table 1 shows the mean differences in children’s anxiety at the beginning, during, and at the end of the test.

### 3.2. Math Test Performance

Children’s anxiety level was negatively related to children’s grades on the test in this study, *r* = −0.27, *p* = 0.018. This means that the higher the anxiety of children, the lower their mathematical performance. Also, there was a positive relationship between children’s previous math grades and their performance on the test in this study, *r* = 0.43, *p* ≤ 0.001. Finally, children’s performance on the math test was negatively related to students’ anxiety during, *r* = −0.24, *p* = 0.042, and at the end of the test, *r* = −0.43, *p* = 0.049, as well as to task difficulty, *r* = −0.31, *p* = 0.014.

Data on mathematical performance were analysed with a two-way analysis of covariance (ANCOVA), with test strategy and anxiety level as between-subjects factors, and previous teachers’ grade as the covariate. The main effects of condition, *F*(1, 63) < 1, *p* = 0.969, and the interaction between the condition and children’s anxiety level was not significant, *F*(1, 63) < 1, *p* = 0.590. The covariate, teacher’s grade, *F*(1, 63) = 11.39, *p* ≤ 0.001, and the main effect of anxiety level, *F*(1, 63) = 4.41, *p* = 0.040, were found significant. In both conditions, high anxious students (*M* = 4.06, *SD* = 1.41) performed worse compared to low anxious students (*M* = 5.11, *SD* = 1.72). The means and standard deviations of the math test as a function of condition and children’s anxiety level are illustrated in Table 2.

### 3.3. Perceived Mental Effort

Correlations showed a positive relationship between mental effort and children’s anxiety at the beginning, *r* = 0.28, *p* = 0.031, during, *r* = 0.33, *p* = 0.010, and at the end of the test, *r* = 0.35, *p* = 0.007. Mental effort was also highly positively correlated with task difficulty, *r* = 0.74, *p* ≤ 0.001.

With regard to the mental effort that children invested when completing the test, a two-way ANOVA was conducted with condition and anxiety level as between-subjects factors. Table 2 portrays the means of invested mental effort as a function of condition and anxiety level. Results showed that neither the main effects of test strategy, *F*(1, 63) < 1, *p* = 0.353, and anxiety level, *F*(1, 63) < 1, *p* = 0.911, nor the interaction between condition and anxiety level, *F*(1, 63) < 1, *p* = 0.904, were significant.

### 3.4. Perceived Task Difficulty

Correlations showed a positive relationship between task difficulty and children’s anxiety at the beginning, *r* = 0.32, *p* = 0.011, during, *r* = 0.41, *p* ≤ 0.001, and at the end of the test, *r* = 0.33, *p* = 0.009. For analysing children’s ratings regarding the task difficulty of the test, a two-way ANOVA was conducted with condition and anxiety level as between-subjects factors. Table 2 depicts the means in task difficulty as a function of condition and anxiety level. There were no significant main effects of condition, *F*(1, 58) < 1, *p* = 0.984, and anxiety level, *F*(1, 58) < 1, *p* = 0.476, nor a significant interaction effect between condition and anxiety level, *F*(1, 58) = 3.33, *p* = 0.073.

## 4. Discussion

This study aimed to explore the effects of a short physical activity break in the classroom on students’ test anxiety and math test performance. The first hypothesis that the high-anxious students would perform worse than low-anxious students on math performance was supported by the data. This finding is in line with previous literature demonstrating the negative effects of anxiety on test performance [3,61,62]: high anxious students, regardless of the condition, were particularly anxious at the beginning and during the test, while they also had lower test scores than the low anxious students. However, we found no support for the second hypothesis, supporting that students performing the 10-minute activity break before the math test would show lower anxiety levels in general, and would outperform the control group in their academic performance.

A possible explanation for this null result is that our manipulation might not have elicited the anxiety experienced when taking real exams. Indeed, the anxiety scale scores were < 5 (on a scale of 1–9) at all time points in all groups, which might be a tolerable level for working memory to function properly (i.e., no overload). We suggest that high anxiety levels have detrimental effects, especially on working memory functioning and (academic) performance. In the current study, there was a roughly equal number of low and high anxious students in each class. In line with numerous studies, a negative relation between self-reported anxiety and performance on working memory tasks was revealed (for a meta-analysis and review, see [63]). Previous research has shown that high anxious participants’ working memory is occupied by threatening thoughts [63], which would otherwise be needed to perform well during complex tasks. As a result, cognitive load in high anxious students is also expected to be very high [64,65].

In general, working memory load moderates late attentional bias in social anxiety [66]. For instance, when working memory load is high, attention is difficult to be disengaged from the causes of threat. In contrast, if working memory load is low, avoidance can occur [66,67]. In the present study, students’ anxiety levels may also have had an impact on their perceived task difficulty and mental effort invested. Although not statistically significant, high anxious students in both groups may have considered the math test as more complex and may have experienced higher mental effort compared to low anxious students, also affecting the effort they put on achieving high in the test and in turn, their mathematical performance.

In fact, Capa, Audiffren, and Ragot (2008) looked at the effects of achievement motivation and task difficulty on invested mental effort in an adult population [68]. Based on their answers in motivation questionnaires, participants were categorised into having a motive to achieve success (focusing on preference for difficult tasks; “approach-driven” group), or a motive to avoid failure (“avoidance-driven” group). In addition, participants completed a visual working memory search task with varying levels of difficulty. Results showed that approach-driven participants had faster reaction times especially during the difficult task, than avoidance-driven participants. As expected, perceived difficulty and mental effort increased as the task became more difficult. However, approach-driven participants showed better performance than avoidance-driven participants with the same amount of mental effort invested, as shown by decreased cardiovascular reactivity during difficult tasks and mean reaction times of approach-driven participants.

Finally, Yerkes and Dodson (1908) suggested a u-shape function between performance and arousal [69]. As such, optimal levels of cognitive performance can be achieved with balanced levels of anxiety. Accordingly, very low levels of anxiety and mental effort invested would deteriorate cognitive performance as much as very high levels of anxiety and mental effort invested. Nevertheless, effective strategies are needed to deal with students’ emerging anxiety during test conditions, along with its negative consequences.

Although we believe that the students’ anxiety levels during our experiment were lower than during official school examinations, high anxious students performed less well than low anxious students. Possibly, intervention studies involving physical activity breaks could be used before test examinations, and this might be able to depict changes in children’s anxiety levels without impeding test performance. Future research investigating the mediating role of physical activity and the moderating role of test anxiety on test performance could provide insight in the mechanisms of this relationship. Systematically manipulating arousal (e.g., anxiety and cognitive exertion) during physical activity tasks is indispensable for provoking cognitive improvements.

In order to shed light on whether participants experience threat or challenge, physiological measures of stress can also be included in future research. For example, Seery (2011) describes patterns of four cardiovascular measurements that can distinguish between physiological threat or challenge responses; heart rate (speed of beats), ventricular contractility (strength of beats), total peripheral resistance (net constriction vs. dilation of the blood vessels) and cardiac output (amount of blood pumped through the body per minute) [70]. Physiological changes associated with challenge may be similar to those associated with aerobic exercise [14]. It would be interesting in the future to investigate whether these physiological patterns occur during test anxiety and how physical activity might change these patterns in more adaptive ones.

Contrary to our hypotheses, we did not find any effect of acute physical activity on students’ anxiety and math test performance. We propose several explanations for these null findings: firstly, the current intervention included one bout of 10 minutes of moderate-to-vigorous physical activity. Possibly, because anxiety is a more generalised and sometimes chronic condition, multiple bouts of physical activity or higher intensity may be required. According to the cross-stressor-adaptation hypothesis [71], psychosocial stress responses such as increased blood cortisol, heart rate and respiration can occur after exposure to physical stress (e.g., vigorous exercise).

However, a physiological adaptation (i.e., hormonal regulation) of the body during physical exercise can be applied also to psychosocial stressors, in turn improving mental health [72]. Currently, there is strong evidence of the benefits of physical activity for children’s and adolescents’ mental health, mainly in depression, anxiety, self-esteem and cognitive functioning [32]. Hence, it can be argued that higher intensity physical activity that improves health-related fitness (i.e., muscular fitness, cardiorespiratory fitness, and body composition) might be needed to elicit neurobiological changes in cognitive and academic performance.

In fact, Chang and colleagues (2012) suggested that heart rate reaching 70%–85% during acute bouts of moderate-to-vigorous physical activity can be linked with positive changes in cognitive performance. In addition, vigorous intensity physical activity appears to produce stronger mental health benefits than activity of light and moderate intensity [73]. The current study did not measure intensity levels of physical activity. Future research needs to use objectively measured physical activity (i.e., accelerometers) in order to accurately report on the intensity levels [74]. This assumption is also confirmed by results of a recent study, in which no difference between the high and low physical activity groups was found on elementary school children’s cognitive performance after an acute single session of physical activity [75]. The authors also concluded that the duration and intensity of physical activity might not have been high enough to elicit an immediate cognitive response. Alternatively, combining physical activity with counselling before the test may produce a reduction in students’ anxiety levels. Apart from the evident benefits of physical activity on mental health, current research has found that counselling is an effective strategy for decreasing specifically test anxiety in students [76].

Overall, the findings of this research contribute to the literature of acute effects of physical activity on mental health, including cognition and level of anxiety. Nevertheless, there are some limitations that need to be acknowledged: Firstly, the current experiment, apart from the limited number of participants, did not involve a real test, and therefore might not have evoked the anxiety response we would have seen in a real test situation in the high anxious group. Adding to this point, the novelty and enjoyment of both tasks (physical activities and vocabulary game) may have also contributed to lower anxiety levels at the beginning of the test. It would be interesting in the future to test the same hypotheses with a more anxiety-evoking manipulation. Another limitation is that in the current set-up, we were not able to distinguish math anxiety, a specific anxiety for mathematical problems, from test anxiety (i.e., anxiety for tests in general) [77]. It is possible that the current manipulation would have been more sensitive when high and low anxious participants were selected on the basis of levels of math anxiety, instead of test anxiety in general. Future studies should be more specific on comparing high versus low math anxious students. Finally, future studies should also aim to replicate the current design across different countries, such as Portugal, Greece, and Spain, where students struggle and underperform more in mathematics compared to Australian students, as shown by the Program for International Student Assessments [78].

## 5. Conclusions

This study pinpointed that using a physical activity break before test examination does not deteriorate test anxiety and math test performance. Even if we are not sure if our manipulation was enough to induce test anxiety, we believe that it could be used as a strategy that could potentially provide students with potential cognitive, psychological and physical benefits. Longer-scale studies might be able to reveal the potential of this approach to students’ long-term wellbeing.

## Figures and Tables

**Table 1:** Means (SD) of children’s perceived anxiety at the beginning, during, and at the end of the test as a function of condition and children’s anxiety level.

	Activity Break Condition		Control Condition	
Anxiety	Low Anxious	High Anxious	Low Anxious	High Anxious
Start of the test	3.00 (2.00)	4.67 (2.35)	2.86 (2.38)	3.92 (2.57)
During the test	2.67 (1.76)	4.67 (1.85)	2.50 (2.34)	4.54 (2.73)
End of the test	3.00 (2.30)	3.76 (2.40)	2.86 (2.82)	3.17 (2.50)

**Table 2:** Means (SD) of math performance, mental effort, and task difficulty as a function of condition and children’s anxiety level.

	Activity Break Condition		Control Condition	
	Low Anxious	High Anxious	Low Anxious	High Anxious
Math performance	5.23 (1.64)	4.00 (1.39)	5.00 (1.85)	4.12 (1.47)
Mental effort	5.12 (1.45)	5.22 (1.56)	4.78 (1.75)	4.78 (1.57)
Task difficulty	4.35 (0.98)	5.28 (1.29)	5.02 (1.44)	4.62 (1.75)
